# The genome sequence of the Dracula fish, *Danionella dracula *(Britz, Conway & Rüber, 2009)

**DOI:** 10.12688/wellcomeopenres.21117.1

**Published:** 2024-04-12

**Authors:** Lukas Rüber, Ralf Britz, Kevin Conway, Iliana Bista, Shane McCarthy, Jonathan Wood, Michelle Smith, Karen Oliver, Kerstin Howe, Richard Durbin

**Affiliations:** 1 Naturhistorisches Museum Bern & Institute of Ecology and Evolution, University of Bern, Bern, Switzerland; 2 Senckenberg Naturhistorische Sammlungen Museum fur Tierkunde Dresden, Dresden, Saxony, Germany; 3 Texas A&M University, College Station, Texas, USA; 4 LOEWE Centre for Translational Biodiversity Genomics, Frankfurt, Germany; 5 Senckenberg Research Institute, Frankfurt, Germany; 6 Tree of Life, Wellcome Sanger Institute, Hinxton, England, UK; 7 University of Cambridge, Cambridge, England, UK

**Keywords:** Danionella dracula, Dracula fish; genome sequence, scaffold-level, Cypriniformes

## Abstract

We present a genome assembly from an individual *Danionella dracula* (the Dracula fish; Chordata; Actinopterygii; Cypriniformes; Danionidae; Danioninae). The genome sequence is 665.21 megabases in span. This is a scaffold-level assembly, with a scaffold N50 of 10.29 Mb.

## Species taxonomy

Eukaryota; Metazoa; Eumetazoa; Bilateria; Deuterostomia; Chordata; Craniata; Vertebrata; Gnathostomata; Teleostomi; Euteleostomi; Actinopterygii; Actinopteri; Neopterygii; Teleostei; Osteoglossocephalai; Clupeocephala; Otomorpha; Ostariophysi; Otophysi; Cypriniphysae; Cypriniformes; Cyprinoidei; Danionidae; Danioninae; *Danionella*; *Danionella dracula* ( Britz *et al.*, 2009)

## Background

**Figure 1.**
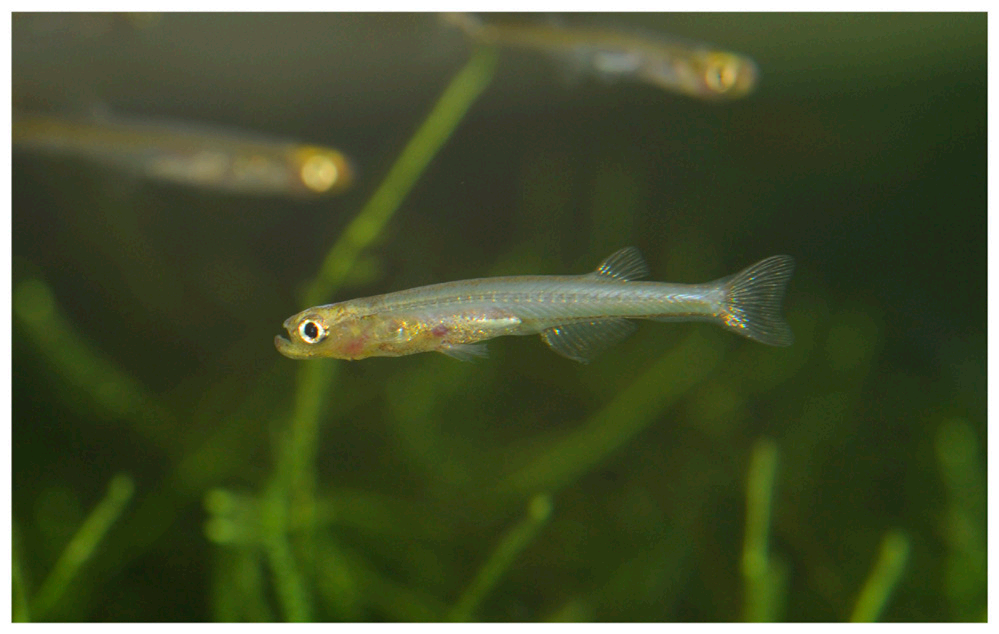
Image of *Danionella dracula* (Photograph by Ralf Britz).

*Danionella dracula* ( Figure 1) is a miniature, transparent freshwater fish from streams around Sha Du Zup, Kachin State, in northern Myanmar with a maximum recorded size of 16.7 mm standard length ( Britz *et al.*, 2009). It forms the sister group of the other four miniature species of *Danionella*: *D. translucida* ( Roberts, 1986), *D. mirifica* ( Britz, 2003), *D. priapus* ( Britz, 2009) and *D. cerebrum* ( Britz *et al.*, 2021). *Danionella dracula* separated from its closest relatives about 29.5 mya ( Britz *et al.*, 2009).

*Danionella dracula* has recently emerged as a model organism for neurophysiological research ( Tatarsky *et al.*, 2022), along with *D. cerebrum* ( Schulze *et al.*, 2018), previously misidentified as *D. translucida* ( Britz *et al.*, 2021). A hybrid genome assembly for *D. cerebrum* has previously been published ( Kadobianskyi *et al.*, 2019), and we complement this with publication of the *D. dracula* genome here.

*Danionella dracula* shows a number of highly unusual features that have been the prerequisite for its establishment as a neurophysiological model organism. The skeleton of *Danionella dracula* is characterised by loss and reduction of 61 bones, bone parts or cartilages ( Britz & Conway, 2016), including the frontals and parietals, which form the skull roof in other bony fishes. *Danionella dracula* is one of the most developmentally truncated fishes, meaning that its anatomy, with few exceptions, resembles that of a larval fish. This organism-wide progenetic condition has facilitated the evolution of several morphological novelties ( Britz & Conway, 2016), most of them sexually dimorphic. The most dramatic sexual dimorphic novelties involve its jaws, with large males developing tooth-like fangs, and its Weberian apparatus, in which males have hypertrophied elements and a bulbous drumming muscle and cartilage ( Britz *et al.*, 2009; Britz & Conway, 2016). Both these character complexes are also exceptions to the organism-wide progenesis, and at least the well-developed Weberian apparatus is the result of striking heterochronic changes during the ontogeny of *D. dracula* ( Conway *et al.*, 2021). While the development of most of the skeleton of *D. dracula* is delayed when compared to the zebrafish and other cypriniforms, that of the components of the Weberian apparatus is greatly accelerated resulting in a larval-looking fish with a well-developed Weberian apparatus used for hearing and sound production ( Conway *et al.*, 2021). This highly unusual combination of an astonishingly transparent body, a reduced skeleton with no skull roof allowing immediate access to one of the smallest vertebrate brains, and a highly developed sound reception and production system combined with a complex behavioural repertoire have made this fish an attractive research subject.

*Danionella dracula* is a member of the Danioninae and a close relative of the zebrafish *Danio rerio*. The Danioninae are an emerging model clade in the field of evo-devo, favoured for analyses of anatomy (especially bone morphology and growth), pigmentation, gene family expansion and phylogeography ( Braasch *et al.*, 2015; Parichy, 2015). Within the Danioninae Sequencing Project we are providing high quality genome assemblies for representatives of this clade to facilitate its use.

## Genome sequence report

**Figure 2.**
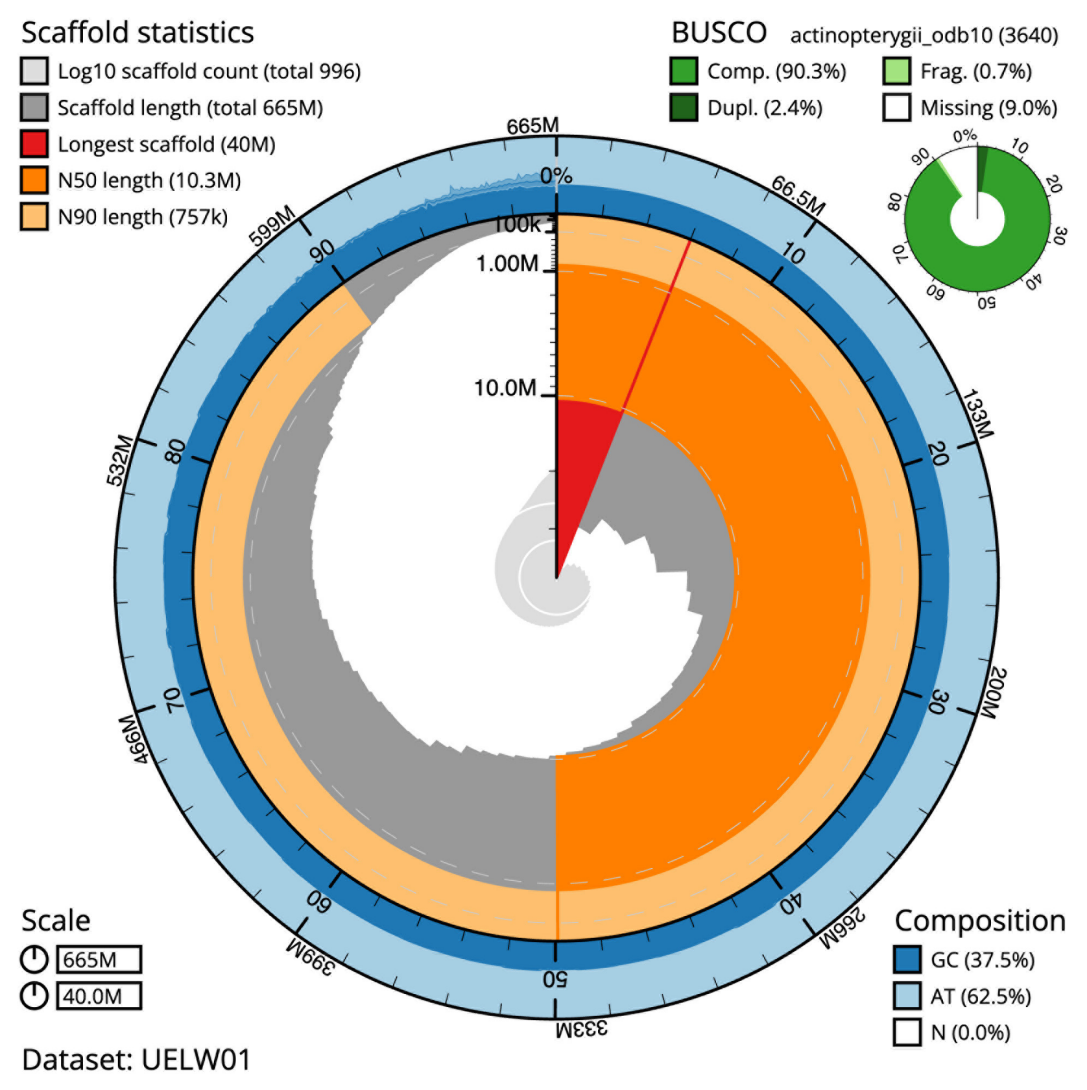
Genome assembly of *Danionella dracula*, fDanDra1.1: metrics. The BlobToolKit Snailplot shows N50 metrics and BUSCO gene completeness. The main plot is divided into 1,000 size-ordered bins around the circumference with each bin representing 0.1% of the 665,208,374 bp assembly. The distribution of scaffold lengths is shown in dark grey with the plot radius scaled to the longest scaffold present in the assembly (39,982,851 bp, shown in red). Orange and pale-orange arcs show the N50 and N90 scaffold lengths (10,287,669 and 757,246 bp), respectively. The pale grey spiral shows the cumulative scaffold count on a log scale with white scale lines showing successive orders of magnitude. The blue and pale-blue area around the outside of the plot shows the distribution of GC, AT and N percentages in the same bins as the inner plot. A summary of complete, fragmented, duplicated and missing BUSCO genes in the actinopterygii_odb10 set is shown in the top right. An interactive version of this figure is available at https://blobtoolkit.genomehubs.org/view/fDanDra1.1/dataset/UELW01/snail.

**Figure 3.**
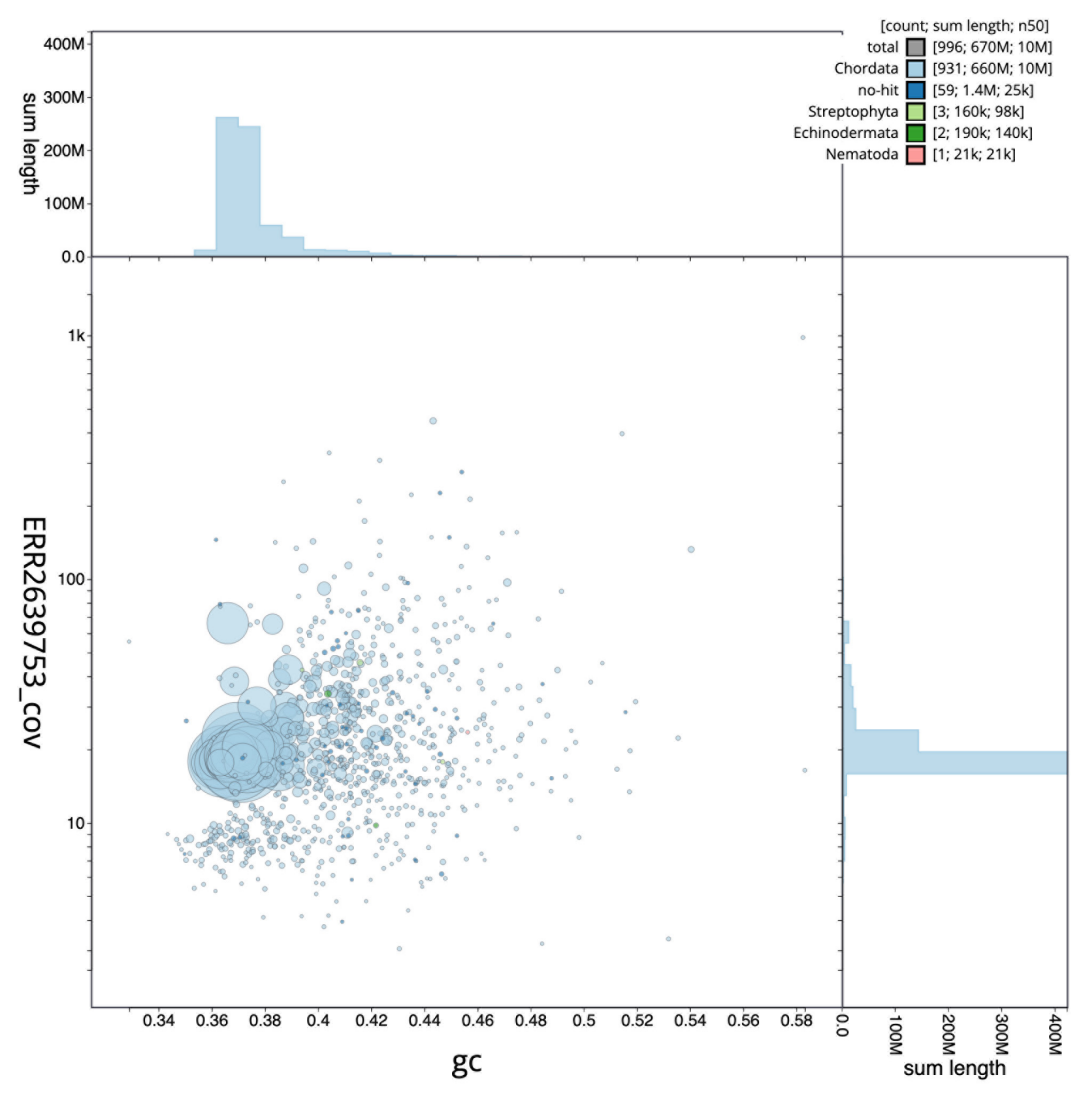
Genome assembly of *Danionella dracula*, fDanDra1.1: BlobToolKit GC-coverage plot. Scaffolds are coloured by phylum. Circles are sized in proportion to scaffold length. Histograms show the distribution of scaffold length sum along each axis. An interactive version of this figure is available at https://blobtoolkit.genomehubs.org/view/fDanDra1.1/dataset/UELW01/blob.

**Figure 4.**
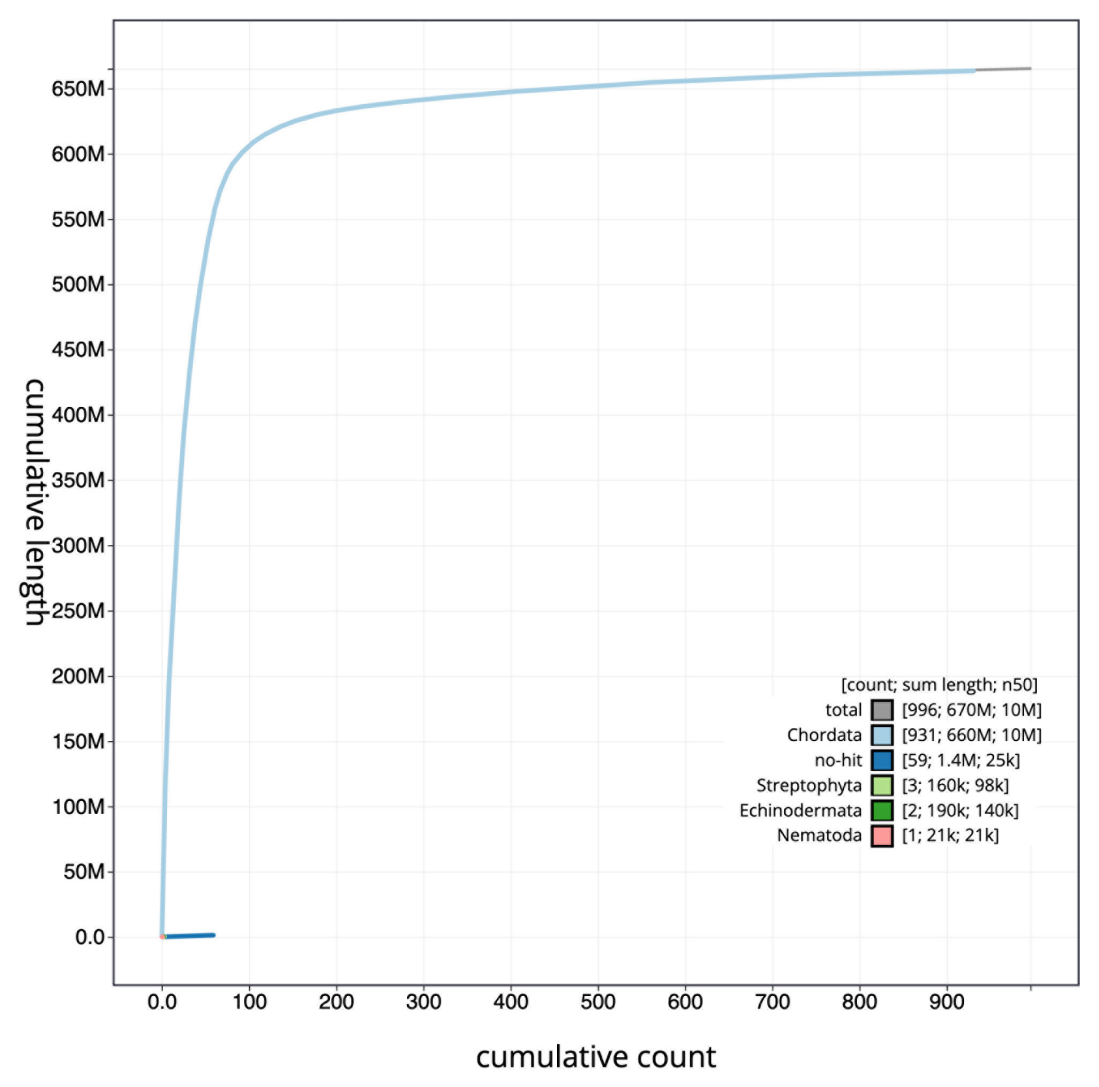
Genome assembly of *Danionella dracula*, fDanDra1.1: BlobToolKit cumulative sequence plot. The grey line shows cumulative length for all scaffolds. Coloured lines show cumulative lengths of scaffolds assigned to each phylum using the buscogenes taxrule. An interactive version of this figure is available at https://blobtoolkit.genomehubs.org/view/fDanDra1.1/dataset/UELW01/cumulative.

The specimen used for the study was obtained through the aquarium trade. The species naturally occurs in a stream near Sha Du Zup between Mogaung and Tanai, Myitkina district, Kachin State, northern Myanmar. A total of 41-fold coverage in Pacific Biosciences single-molecule continuous long reads (CLR) and 104-fold coverage in 10X Genomics read clouds were generated. The final assembly has a total length of 665.21 Mb in 996 sequence scaffolds with a scaffold N50 of 10.29 Mb ( Table 1, Figure 2, Figure 3 and Figure 4).

**Table 1.** Genome data for *Danionella dracula*, fDanDra1.1.

Project accession data	
Assembly identifier	fDanDra1.1
Species	*Danionella dracula*
Specimen	fDanDra1
NCBI taxonomy ID	623740
BioProject	PRJEB27320
BioSample ID	SAMEA104026433
Isolate information	fDanDra1
Assembly metrics	
Consensus quality (QV)	34.6
*k*-mer completeness	98.97%
BUSCO *	C:90.3%[S:87.9%,D:2.4%],F:0.7%,M:9.0%,n:3,640
Raw data accessions	
PacificBiosciences CLR	ERR2639734–ERR2639748
10X Genomics Illumina	ERR2639753–ERR2639756
Genome assembly	
Assembly accession	GCA_900490495.1
Span (Mb)	665.21
Number of contigs	1,611
Contig N50 length (Mb)	2.3
Number of scaffolds	996
Scaffold N50 length (Mb)	10.3
Longest scaffold (Mb)	40

* BUSCO scores based on the actinopterygii_odb10 BUSCO set using v5.3.2. C = complete [S = single copy, D = duplicated], F = fragmented, M = missing, n = number of orthologues in comparison. A full set of BUSCO scores is available at https://blobtoolkit.genomehubs.org/view/fDanDra1.1/dataset/UELW01/busco.

The estimated Quality Value (QV) of the final assembly is 34.6 with *k*-mer completeness of 98.97%, and the assembly has a BUSCO v5.3.2 completeness of 90.3% (single = 87.9%, duplicated = 2.4%), using the actinopterygii_odb10 reference set ( *n* = 3,640).

Metadata for spectra estimates and sequencing runs can be found at https://links.tol.sanger.ac.uk/species/623740.

## Methods

### Sample acquisition and nucleic acid extraction

The *Danionella dracula* specimen used for the genome assembly (BioSample ID SAMEA104026433, individual fDanDra1), was obtained from the laboratory of Ralf Britz. Individuals of *D. dracula* were maintained in an aquarium with the dimensions 80×40×40 cm and fed with brine shrimp nauplii. Specimens were euthanised with an overdose of MS222. Tissue was flash-frozen on dry ice. Two different DNA extraction methods were applied for the fDanDra1 sample. DNA was then extracted from head tissue using a modified version of the MagAttract protocol to increase the yield. HMW DNA was sheared into an average fragment size of 12–20 kb in a Megaruptor 3 system with speed setting 30. Sheared DNA was purified by solid-phase reversible immobilisation using AMPure PB beads with a 1.8X ratio of beads to sample to remove the shorter fragments and concentrate the DNA sample. The concentration of the sheared and purified DNA was assessed using a Nanodrop spectrophotometer and Qubit Fluorometer and Qubit dsDNA High Sensitivity Assay kit. Fragment size distribution was evaluated by running the sample on the FemtoPulse system.

### Sequencing

Pacific Biosciences circular consensus and 10X Genomics read cloud DNA sequencing libraries were constructed according to the manufacturers’ instructions. DNA sequencing was performed by the Scientific Operations core at the WSI on Pacific Biosciences SEQUEL (CLR) and HiSeqX (10X) instruments.

### Genome assembly, curation and evaluation

The assembly fDanDra1.1 is based on PacBio Sequel data, and Illumina HiSeqX data generated from a 10X Genomics Chromium library. An initial PacBio assembly was made using Falcon-unzip ( Chin *et al.*, 2016). The primary contigs were first scaffolded using a wtdbg ( Ruan & Li, 2020) assembly as a synteny guide, then scaffolded further using the 10X data with scaff10x. After using the PacBio data to gap fill with PBJelly ( English *et al.*, 2012) and polishing with Arrow, the assembly was polished again using the 10X Illumina data and FreeBayes ( Garrison & Marth, 2012). Retained haplotigs were identified with purge_haplotigs ( Roach *et al.*, 2018). Finally, the assembly was manually assessed using gEVAL ( Chow *et al.*, 2016).

To assess the assembly metrics, the *k*-mer completeness and QV consensus quality values were calculated in Merqury ( Rhie *et al.*, 2020). This work was done using Nextflow ( Di Tommaso *et al.*, 2017) DSL2 pipelines “sanger-tol/readmapping” ( Surana *et al.*, 2023a) and “sanger-tol/genomenote” ( Surana *et al.*, 2023b). The genome was analysed within the BlobToolKit environment ( Challis *et al.*, 2020) and BUSCO scores ( Manni *et al.*, 2021; Simão *et al.*, 2015) were calculated.

Table 2 lists the relevant software tool versions and sources.

**Table 2.** Software tools: versions and sources.

Software tool	Version	Source
Arrow	GenomicConsensus 2.3.3	https://github.com/PacificBiosciences/GenomicConsensus
bcftools consensus	1.9	http://samtools.github.io/bcftools/bcftools.html
BlobToolKit	4.0.7	https://github.com/blobtoolkit/blobtoolkit
BUSCO	5.3.2	https://gitlab.com/ezlab/busco
Dot	-	https://github.com/dnanexus/dot
falcon_unzip	falcon-kit 1.2.2	https://github.com/PacificBiosciences/FALCON_unzip
FreeBayes	v1.1.0-3-g961e5f3	https://github.com/freebayes/freebayes
gEVAL	N/A	https://geval.org.uk/
Hifiasm	0.12	https://github.com/chhylp123/hifiasm
HiGlass	1.11.6	https://github.com/higlass/higlass
Long Ranger ALIGN	2.2.2	https://support.10xgenomics.com/genome-exome/software/ pipelines/latest/advanced/other-pipelines
Merqury	MerquryFK	https://github.com/thegenemyers/MERQURY.FK
Nucmer	3.9.4alpha	http://mummer.sourceforge.net/
PBJelly		https://github.com/esrice/PBJelly
PretextView	0.2	https://github.com/wtsi-hpag/PretextView
purge_haplotigs	-	https://bitbucket.org/mroachawri/purge_haplotigs/src/master/
sanger-tol/ readmapping	1.1.0	https://github.com/sanger-tol/readmapping/tree/1.1.0
scaff10x	4.2	https://github.com/wtsi-hpag/Scaff10X
wtdbg	-	https://github.com/ruanjue/wtdbg

### Wellcome Sanger Institute – Legal and Governance

The materials that have contributed to this genome note have been supplied by a Tree of Life collaborator. The Wellcome Sanger Institute employs a process whereby due diligence is carried out proportionate to the nature of the materials themselves, and the circumstances under which they have been/are to be collected and provided for use. The purpose of this is to address and mitigate any potential legal and/or ethical implications of receipt and use of the materials as part of the research project, and to ensure that in doing so we align with best practice wherever possible. The overarching areas of consideration are:

•   Ethical review of provenance and sourcing of the material

•   Legality of collection, transfer and use (national and international).

Each transfer of samples is undertaken according to a Research Collaboration Agreement or Material Transfer Agreement entered into by the Tree of Life collaborator, Genome Research Limited (operating as the Wellcome Sanger Institute) and in some circumstances other Tree of Life collaborators.

## Data Availability

European Nucleotide Archive: Dracula fish *Danionella dracula* genome assembly fDanDra1.1. Accession number PRJEB27320; https://identifiers.org/ena.embl/PRJEB27320 ( Wellcome Sanger Institute, 2018). The genome sequence is released openly for reuse. The *Danionella dracula* genome sequencing initiative is part of the Vertebrate Genomes Project. All raw sequence data and the assembly have been deposited in INSDC databases. Raw data and assembly accession identifiers are reported in Table 1.
