# Supplementary material for: The genome sequence of the Dracula fish, Danionella dracula (Britz, Conway & Rüber, 2009)
Source: Wellcome Open Res. 2024 Apr 12;9:194. [Version 1] doi: 10.12688/wellcomeopenres.21117.1 (PMC11367074; doi:10.12688/wellcomeopenres.21117.1)
Supplement: Peer review history

# Reviewer report: Reviewer response for version 1

**DOI:** 10.21956/wellcomeopenres.23357.r93348

**Published:** 2024-09-26

**Authors:** Kathryn Elmer

**Affiliations:** 1 University of Glasgow, Scotland, UK

This resource is developed for a fish that is of considerable interest for the basis of its developmental  and morphological features and as a neurophysiological model. 

 The reference genome is well described and clearly presented. There is logical flow and appropriate level of detail. The  approaches applied are well motivated and sufficiently comprehensive for the purpose of releasing this useful resource. I have no further requests for clarification and I look forward to seeing this taken up by the community

Are sufficient details of methods and materials provided to allow replication by others?

Yes

Is the rationale for creating the dataset(s) clearly described?

Yes

Are the datasets clearly presented in a useable and accessible format?

Yes

Are the protocols appropriate and is the work technically sound?

Yes

Reviewer Expertise:

evolutionary biology

I confirm that I have read this submission and believe that I have an appropriate level of expertise to confirm that it is of an acceptable scientific standard.

# Reviewer report: Reviewer response for version 1

**DOI:** 10.21956/wellcomeopenres.23357.r93356

**Published:** 2024-09-01

**Authors:** Chenhong Li

**Affiliations:** 1 Shanghai Ocean University, Shanghai, China

Dracula fish is a interesting fish the only one with fangs in carp species. The data generated are very useful. All analyses and information extraction were done appropriately. The species itself is unique. The genome resource data collected here will be valuable to further investigate this creature and to serve the purpose of using it as a model organism.

Are sufficient details of methods and materials provided to allow replication by others?

Yes

Is the rationale for creating the dataset(s) clearly described?

Yes

Are the datasets clearly presented in a useable and accessible format?

Yes

Are the protocols appropriate and is the work technically sound?

Yes

Reviewer Expertise:

Phylogenetics, comparative genomics, fish

I confirm that I have read this submission and believe that I have an appropriate level of expertise to confirm that it is of an acceptable scientific standard.

# Reviewer report: Reviewer response for version 1

**DOI:** 10.21956/wellcomeopenres.23357.r88235

**Published:** 2024-07-30

**Authors:** Rohit Kolora

**Affiliations:** 1 Alector Therapeutics, South San Francisco, California, USA

In the manuscript titled "The genome sequence of the Dracula fish, *Danionella dracula* ( *Britz, Conway & Rüber*, 2009)" by *Rüber* et al. the authors sequenced and assembled a scaffold-level genome of Dracula fish. They describe the importance of this resource as a model of neurophysiological research, and improve the genome quality compared to the previously published hybrid assembly. The manuscript is well written and the authors provide most relevant details. The workflow for the assembly assessment is cited including the list of tools/software for the assembly. However, the code/scripts used to generate the actual assembly is missing in the references and availability statement. It would be of great help to the research community for reproducibility if these details (data-specific parameters) can be provided through relevant Zenodo/Git repositories. If default parameters were used for all the tools, please mention it specifically in the methods section.

Are sufficient details of methods and materials provided to allow replication by others?

No

Is the rationale for creating the dataset(s) clearly described?

Yes

Are the datasets clearly presented in a useable and accessible format?

Yes

Are the protocols appropriate and is the work technically sound?

Yes

Reviewer Expertise:

Genomics, Bioinformatics

I confirm that I have read this submission and believe that I have an appropriate level of expertise to confirm that it is of an acceptable scientific standard.
